# A Multifactorial Model for Building Professional Confidence in New Graduate Nurses: Integrative Review of Influential Strategies and Contexts

**DOI:** 10.1155/jonm/6942823

**Published:** 2026-08-23

**Authors:** Justin Fontenot, Fuqin Liu, Jincy Immanuel

**Affiliations:** ^1^ Program of Nursing, Tulane University School of Medicine, New Orleans, Louisiana, USA, tulane.edu; ^2^ College of Nursing, Texas Woman’s University, Denton, Texas, USA, twu.edu; ^3^ School of Nursing and Midwifery, Western Sydney University, Sydney, Australia, westernsydney.edu.au

## Abstract

**Aim:**

To synthesize evidence on strategies and contextual factors that influence confidence development among newly graduated nurses in acute care settings and to propose an integrated conceptual framework to guide practice, policy, and research.

**Design:**

Integrative literature review.

**Methods:**

This integrative review followed Whittemore and Knafl’s framework and PRISMA 2020 reporting guidance. Two reviewers screened studies, extracted data, and applied design‐specific JBI critical appraisal tools. Disagreements were resolved by consensus and consultation with a third reviewer. Data were synthesized through constant comparison and coded by outcome tier (direct confidence/self‐efficacy, related capacity, and proxy transition outcomes).

**Data Sources:**

Five databases (Academic Search Complete, PubMed/Medline, CINAHL Complete, and PsycINFO) were searched from inception through June 30, 2024, with an updated search in May 2025.

**Results:**

Forty studies met the inclusion criteria, representing qualitative, quantitative, and mixed methods designs. Five mechanism‐based domains were identified: support, skill development, reflection, integration, and organizational culture. Organizational culture functions as a conditioning context that can strengthen or constrain mentorship, skill development, reflection, and integration. These findings informed a conceptual framework illustrating the cyclical and dynamic relationships between individual strategies and organizational conditions.

**Conclusion:**

Confidence‐building is a multifaceted and ongoing process influenced by mentorship, targeted training, reflection, team integration, and organizational culture. A comprehensive approach is necessary to help newly graduated nurses transition successfully into acute care practice.

**Implications for Nursing Management:**

Nurse managers play a central role in shaping the conditions that allow newly graduated nurses to develop professional confidence during transition to practice. The findings from this review suggest that confidence‐building strategies are most effective when supported by psychologically safe unit cultures, protected preceptor time, constructive feedback, manageable workloads, and structured opportunities for reflection and team integration. Rather than treating confidence as an individual trait, nursing management should approach it as a workforce outcome influenced by staffing, leadership practices, mentorship infrastructure, and organizational socialization. Strengthening these conditions may improve transition experiences, support retention, and contribute to safer, more stable acute care environments.

## 1. Introduction

Newly graduated nurses (NGNs) face a challenging transition as they move from educational preparation and supervised clinical learning into accountable professional practice [1]. Although national licensure and registration systems are designed to establish entry‐level competence, international evidence continues to show that the first 1‐2 years of acute care practice are marked by uncertainty, transition shock, workload pressures, and variable access to support [2]. Across jurisdictions, entry to practice is regulated through licensure or registration systems that vary in terminology and process but share a common purpose: protecting the public by establishing minimum competence for safe practice. However, regulatory entry does not ensure immediate professional confidence or contextual readiness for complex acute care environments.

In the context of global nursing shortages, early‐career attrition has substantial implications for workforce stability, patient safety, and leadership planning. In the United States (U.S.), the U.S. Bureau of Labor Statistics estimates that about 177,400 new nurses enter the workforce each year [3]. However, early career attrition remains a major concern. For example, a workforce report by NSI Nursing Solutions [4] found that 23.8% of new nurses leave their jobs within the first year, and nearly half leave within 2 years. U.S. workforce reports provide only one example of this challenge, but the transition‐to‐practice problem is international and shaped by local staffing systems, orientation structures, and professional cultures. These statistics highlight an urgent need to understand the reasons behind early departures and dissatisfaction among NGNs.

Multiple interventions clearly influence confidence and self‐efficacy in NGNs: structured transition programs and mentorship [5, 6], simulation and targeted skills training [7], and purposeful strategies to boost self‐confidence during the initial months of practice [8]. Because confidence can be cultivated and is closely related to daily clinical activities, it deserves special attention as both an outcome and a lever within transition programs. Much of the existing research has focused on transition shock, which is a period of emotional, professional, and psychological upheaval as NGNs shift from students to practicing nurses [9, 10]. In response, many healthcare organizations have implemented interventions, such as nurse residency programs, structured preceptorships, and peer support initiatives. Although these approaches aim to ease the transition, ongoing turnover suggests that current efforts may not fully address the root causes of early‐career departures.

Previous research has rightfully highlighted transition shock; however, few studies focus on how specific strategies boost confidence and how contextual factors (such as culture and team integration) affect those outcomes. This comprehensive review fills that gap by (a) summarizing confidence‐building strategies for NGNs in acute care, (b) explaining how organizational culture influences confidence‐building, and (c) introducing a multifactorial model that places confidence within a cyclical process connecting support, skill development, reflection, and integration [5–13].

### 1.1. Conceptual and Theoretical Foundation

For this review, professional confidence is defined as an NGN’s context‐sensitive belief in their ability to apply nursing knowledge, exercise clinical judgment, communicate, and fulfill patient care responsibilities in real‐world practice settings. Confidence overlaps conceptually with self‐efficacy but is not identical to competence. Self‐efficacy refers to one’s belief in the capability to organize and execute actions required to achieve desired performance, whereas competence reflects the demonstrated integration of knowledge, skills, attitudes, and judgment in practice [14, 15]. Transition outcomes include broader indicators such as satisfaction, role adaptation, and turnover intention. Therefore, this review treats confidence and self‐efficacy as direct proximal outcomes and treats competence, readiness, satisfaction, and transition shock as related but indirect indicators.

Bandura’s self‐efficacy theory provides the psychological basis for the resulting model [16]. Confidence develops through mastery experiences, vicarious learning, verbal persuasion, and interpretation/regulation of affective and physiological states. In NGN transition, these sources map onto repeated clinical task mastery, observation of preceptors and peers, constructive feedback, and emotionally safe debriefing after uncertainty or error. This theoretical framing explains why support, skill development, reflection, and integration operate as confidence‐building mechanisms rather than isolated strategies.

From a nursing management perspective, organizational culture shapes the accessibility and credibility of sources of self‐efficacy. Unit norms, leadership practices, workload, psychological safety, and structural empowerment determine whether NGNs receive timely feedback, protected practice opportunities, help‐seeking without humiliation, and progressive autonomy. Thus, culture is conceptualized here as a conditioning context: It may amplify confidence‐building strategies when it supports learning and belonging and may constrain them when workload, incivility, or poor preceptor continuity limit safe participation.

## 2. The Review

### 2.1. Aims and Objectives

Given the role of confidence in shaping NGNs’ transition experience, this integrative review examined the strategies and contextual factors that influence the development of confidence among NGNs in acute care settings. The research question guiding this integrative review was:

• What strategies, mechanisms, and contextual conditions contribute to professional confidence development among newly graduated registered nurses during their first 1‐2 years of transition into acute care settings?

The objectives of the review were to:

1. Synthesize the evidence on confidence‐building strategies for NGNs in acute care settings.
2. Explain how organizational culture and unit‐level conditions shape, strengthen, or constrain confidence‐building strategies.
3. Identify key gaps in the literature to inform future theory‐building and practice‐based research.

### 2.2. Design

This integrative review followed the framework provided by Whittemore and Knafl [17]. This approach was the most suitable because the framework enables a comprehensive, systematic synthesis of research evidence across diverse methodologies. This approach was selected to address the complexity of the research question. The framework encompasses five stages: problem identification, literature search, data evaluation, data analysis, and presentation. The PRISMA reporting guidelines were used to guide the reporting of the findings in this integrative review. No protocol was registered for this integrative review; this is acknowledged as a limitation. A completed PRISMA checklist was prepared for submission as supporting information.

### 2.3. Eligibility Criteria

Studies were eligible if they examined confidence, self‐confidence, or self‐efficacy directly or examined a clearly related transition‐to‐practice construct with explicit implications for confidence development. During synthesis, studies were separated into direct confidence/self‐efficacy evidence and proxy evidence. Proxy studies were used to contextualize mechanisms but not to make direct claims about confidence effects. Additional inclusion criteria for this integrative review were as follows: (1) primary studies that focused on NGNs within their first 1‐2 years of practice, (2) research and reports conducted in acute care settings, and (3) empirical studies utilizing qualitative, quantitative, or mixed methods. Exclusion criteria were as follows: (1) studies not in English, (2) studies focusing on nurses beyond the first 1‐2 years of practice, (3) studies conducted in nonacute care settings, and (4) gray literature publications and previously published literature reviews. The review was limited to English‐language and retrievable full‐text empirical studies because the team did not have translation resources and because full methodological appraisal required complete reports. Gray literature was excluded to maintain focus on peer‐reviewed empirical evidence; this choice may have increased publication bias and is noted as a limitation.

### 2.4. Information Sources

The following databases were searched using a predefined search strategy: Academic Search Complete, PubMed, CINAHL Complete, PsycINFO, and Medline. The timeframe covered was from inception to May 2025. Search results were exported to Covidence by the first author. The search strategy was developed by the first author in consultation with a medical librarian and reviewed by the coauthors. Title/abstract screening, full‐text review, extraction, and appraisal were completed by at least two members of the research/dissertation team, with disagreements resolved through consensus discussion and, when needed, full‐team review.

### 2.5. Search Strategy

A list of keywords and related concepts was created after refining the research question guiding this review. Since integrative reviews often incorporate noninterventional studies (e.g., qualitative research, policy papers, and theoretical frameworks), the Population, Concept, and Context (PCC) framework is more suitable than the PICO framework, which is geared toward clinical interventions and comparative effectiveness research. Population: NGNs, new graduate nurses, novice nurses, recently licensed nurses, and entry‐level nurses. Concepts: clinical decision‐making, clinical competence, clinical judgment, clinical reasoning, and decision‐making confidence. Context: first‐year, postlicensure, early career, initial year, first 12 months, techniques, strategies, methods, approaches, and interventions. After the keywords were checked and refined against the research question and the review’s aims and objectives, a medical librarian was consulted to design the search strategy. Table 1 lists the final search strategies for each database.

**TABLE 1:** Boolean search syntax by database.

Database name	Search strategy	Limiters
Academic search complete	(newly graduated nurses OR new graduate nurses OR novice nurses OR recently licensed nurses OR entry‐level nurses) AND (clinical decision making OR clinical competence OR clinical judgment OR clinical reasoning OR decision‐making confidence OR self‐confidence OR professional confidence) AND (first year OR post‐licensure OR early career OR initial year OR first 12 months) AND (techniques OR strategies OR methods OR approaches OR interventions)	Full text, English
PubMed	((“newly graduated nurses” OR “new graduate nurses” OR “novice nurses” OR “recently licensed nurses” OR “entry‐level nurses”) [Title/Abstract]) AND ((“clinical decision making” OR “clinical competence” OR “clinical judgment” OR “clinical reasoning” OR “decision‐making confidence” OR “professional confidence”) [Title/Abstract]) AND ((“first year” OR “post‐licensure” OR “early career” OR “initial year” OR “first 12 months”) [Title/Abstract]) AND ((“techniques” OR “strategies” OR “methods” OR “approaches” OR “interventions”) [Title/Abstract])	Full text, English
CINAHL Complete	(newly graduated nurses OR new graduate nurses OR novice nurses OR recently licensed nurses OR entry‐level nurses) AND (clinical decision making OR clinical competence OR clinical judgment OR clinical reasoning OR decision‐making confidence OR self‐confidence OR professional confidence) AND (first year OR post‐licensure OR early career OR initial year OR first 12 months) AND (techniques OR strategies OR methods OR approaches OR interventions)	Full text, English
PsycINFO	(newly graduated nurses OR new graduate nurses OR novice nurses OR recently licensed nurses OR entry‐level nurses) AND (clinical decision making OR clinical competence OR clinical judgment OR clinical reasoning OR decision‐making confidence OR self‐confidence OR professional confidence) AND (first year OR post‐licensure OR early career OR initial year OR first 12 months) AND (techniques OR strategies OR methods OR approaches OR interventions)	Full text, English
Medline	(newly graduated nurses OR new graduate nurses OR novice nurses OR recently licensed nurses OR entry‐level nurses) AND (clinical decision making OR clinical competence OR clinical judgment OR clinical reasoning OR decision‐making confidence OR self‐confidence OR professional confidence) AND (first year OR post‐licensure OR early career OR initial year OR first 12 months) AND (techniques OR strategies OR methods OR approaches OR interventions)	Full text, English

### 2.6. Study Selection Process

Once the search results were uploaded into Covidence, the articles were initially screened by title and abstract. After this initial screening, the full texts of each article were uploaded into the system for further review. Two independent reviewers screened titles and abstracts against the eligibility criteria. Articles marked include or unsure by either reviewer advanced to full‐text screening. Full texts were reviewed independently by two reviewers. Disagreements were resolved through discussion; persistent disagreements were adjudicated by the third reviewer. Interrater reliability statistics were not calculated, which is reported as a methodological limitation. The conceptual distinctions described in the Introduction guided screening and extraction. Each included study was classified as direct confidence/self‐efficacy evidence, related capacity evidence, or proxy transition evidence.

### 2.7. Data Collection and Analysis

The researcher conducted a quality analysis using the assessment instruments provided by the Joanna Briggs Institute (JBI) [18]. Design‐specific JBI tools were used: the JBI checklist for analytical cross‐sectional studies, checklist for quasiexperimental studies, checklist for randomized controlled trials, and checklist for qualitative research. Mixed‐methods studies were appraised using the relevant JBI checklists for each component, when possible. Quality ratings were assigned as follows: high = most JBI items met with no major validity concerns; moderate = several items unclear or unmet, but findings generally interpretable; low = multiple unmet items that substantially limited confidence in the findings; unclear = insufficient reporting to judge key quality indicators. Ratings were not used to exclude studies but were used to weigh the interpretation.

The analysis followed Whittemore and Knafl’s stages: data reduction, data display, data comparison, conclusion drawing, and verification. The first cycle of coding identified confidence‐building strategies, outcomes, level of action (individual, dyadic, unit, and organizational), and outcome tier. Focused coding, then grouped strategies by mechanism: social persuasion/support, mastery/practice, reflective meaning‐making, professional integration, and organizational conditioning. Credibility was supported through team coding meetings, constant comparison across study designs and countries, negative case searching, and rechecking themes against quality‐rated evidence. Dependability was supported by an audit trail comprising the extraction matrix, coding notes, quality appraisal decisions, and consensus notes.

## 3. Findings

### 3.1. Study Characteristics

**FIGURE 1:**
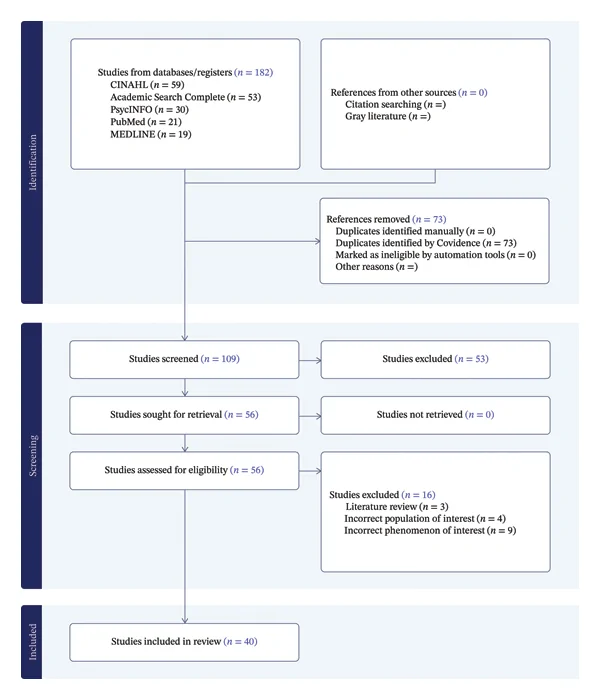
PRISMA flowchart.

Forty studies were included, representing 16 countries and one multinational sample, with publication years ranging from 2004 to 2025. The sample included 11 quantitative, 21 qualitative, and 8 mixed‐methods studies, representing 3065 new graduate nurses. The PRISMA flow diagram presents screening and exclusion details noted in Figure 1.

### 3.2. Quality Appraisal

Several studies did not provide sufficient detail to assess the methods, design, and potential bias entirely and were, therefore, marked as unknown. Following the data evaluation stage outlined by Whittemore and Knafl, the methodological quality was incorporated into evidence synthesis using four categories: high (HQ), moderate (MQ), low (LQ), and unclear (UQ) quality (UQ = insufficient reporting to assess key items). During data display and comparison, HQ evidence served as the main confirmation, MQ as supporting evidence, and LQ/UQ provided context. A pattern or theme was considered a synthesis‐level finding only if supported by at least two HQ/MQ studies across at least two settings. Patterns found only in LQ/UQ were labeled “tentative” and kept for hypothesis generation. A qualitative sensitivity analysis was performed by rereviewing the findings limited to HQ/MQ studies. Table 2 presents the analytic synthesis of confidence‐building domains, mechanisms, evidence strength, and implications for nursing management. Detailed study characteristics, evidence tiers, domain mapping, and quality appraisal ratings are provided in Supporting Table S1.

**TABLE 2:** Analytic synthesis of confidence‐building domains, evidence strength, and nursing management implications.

Domain	Confidence‐building mechanism	Subcomponents	Nature of evidence	Strength and cautions	Implications for nursing management
Support	Verbal persuasion, vicarious learning, and social validation help NGNs judge that they can perform safely with guidance.	Mentorship; preceptorship; peer support; constructive feedback; debriefing with experienced nurses.	Direct confidence/self‐efficacy evidence appears in mentoring, simulation, and peer‐support studies; related evidence includes competence, readiness, and retention outcomes.	Strongest when mentorship/preceptorship and feedback are clearly structured. Mostly qualitative and mixed‐methods evidence; several outcomes are confidence‐adjacent rather than direct confidence measures.	Protect preceptor time, train preceptors in feedback, distinguish mentoring from peer support, and create formal peer connection points during the first year.
Skill development	Mastery experiences build confidence when NGNs repeatedly practice clinical judgment, technical skills, and prioritization in safe but realistic conditions.	Simulation; targeted skills training; hands‐on workshops; postgraduate/continuing education; deteriorating‐patient preparation.	Direct confidence evidence is strongest in simulation and self‐confidence studies; proxy evidence includes competence, knowledge, clinical skills, work readiness, and patient‐safety knowledge.	Moderate to strong evidence, but many studies measure competence or readiness rather than confidence directly. Interpretation should separate skill acquisition from confidence development.	Use staged skill progression, simulation for high‐risk events, and continuing education that aligns with unit‐specific risk areas and role expectations.
Reflection	Reflection supports self‐appraisal and affective regulation, helping NGNs convert experience into learning rather than threat.	Structured reflection; guided debriefing; self‐assessment; reflection after errors or difficult clinical situations.	Evidence is primarily qualitative or mixed‐methods; direct confidence evidence is present in selected studies, while other studies report critical thinking, learning, satisfaction, or professional growth.	Moderate evidence. Strong conceptual fit with self‐efficacy theory, but fewer studies isolate reflection as an independent intervention.	Build routine debriefing and reflective self‐assessment into residency, orientation, and postpreceptorship check‐ins rather than leaving reflection to individual initiative.
Integration	Progressive participation in the clinical community helps NGNs move from peripheral participation to credible team membership, strengthening confidence through belonging and role mastery.	Workgroup socialization; task mastery; role transition; team communication; gradual autonomy; role clarity.	Evidence includes direct confidence findings and related outcomes such as work readiness, socialization, competence, and satisfaction with transitional support.	Moderate to strong evidence, especially for socialization and role transition. However, integration is often embedded in broader transition programs and not always measured separately.	Create staged autonomy, explicit role expectations, team inclusion practices, and opportunities for NGNs to be recognized as legitimate contributors.
Organizational culture	Culture functions as a conditioning context that amplifies or constrains other confidence‐building strategies through psychological safety, workload, learning climate, and norms for help‐seeking.	Psychological safety; just culture; inclusive team climate; workload/resource adequacy; antibullying norms; leadership support; professional development climate.	Evidence is largely contextual and indirect, with studies showing that support, socialization, workload, and team climate shape confidence‐related outcomes.	Important but should be framed cautiously: the review supports culture as a contextual moderator/conditioning context rather than a statistically tested moderator across all studies.	Treat confidence as a nursing‐management outcome. Align staffing, preceptor resources, feedback systems, incivility prevention, and learning climate with NGN transition goals.

*Note:* “Direct confidence/self‐efficacy evidence” refers to studies that explicitly measured or reported confidence, self‐confidence, professional confidence, or self‐efficacy. “Related/proxy evidence” refers to studies that measured or described competence, readiness, satisfaction, transition shock, socialization, retention, or other outcomes relevant to confidence development but not equivalent to confidence. Organizational culture should be described as a contextual moderator or conditioning context unless a study statistically tested moderation.

Abbreviation: NGN, newly graduated nurse.

### 3.3. Support

The domain of support represents the interpersonal and structural resources through which NGNs develop confidence during transition to professional practice. Across the included studies, support was not merely an emotional buffer; it served as a mechanism through which NGNs observed competent practice, received guided feedback, tested emerging clinical judgments, and gained reassurance that help was available when uncertainty arose. Viewed through a self‐efficacy lens, supportive relationships contributed to confidence through modeling, verbal persuasion, social validation, and reduced anxiety during early clinical encounters. This distinction is important because support did not build confidence in isolation. Rather, mentorship, preceptorship, peer connections, and feedback appeared to strengthen confidence when embedded in clinical environments that allowed NGNs to ask questions, practice skills, and receive timely guidance without fear of judgment.

Mentorship and preceptorship were the most frequently identified support mechanisms. Several studies showed that mentors and preceptors helped NGNs translate theoretical knowledge into situated clinical judgment by providing supervision, role modeling, and practical coaching during patient care situations [6, 19–28]. These relationships appeared especially important when NGNs encountered unfamiliar procedures, complex patient needs, or uncertainty about prioritization and escalation. In this way, mentorship and preceptorship contributed to confidence by making clinical expectations visible and by allowing NGNs to gradually assume responsibility while maintaining access to an experienced clinician. However, the evidence also suggests that the effectiveness of mentorship depended on the quality, continuity, and accessibility of the relationship. Supportive mentors provided emotional reassurance, acceptance, guidance, and encouragement, whereas inconsistent or poorly structured support left NGNs vulnerable to isolation and uncertainty [24, 28–30].

Peer support emerged as a distinct but complementary source of confidence‐building. Unlike mentorship, which generally positions the experienced nurse as a guide or evaluator, peer support provides normalization, shared problem‐solving, and a sense of belonging for nurses undergoing similar transitions. Included studies described peer networks, group debriefing, and cohort‐based transition activities as important opportunities for NGNs to share challenges, compare experiences, and reduce feelings of loneliness or inadequacy [11, 24, 31–36]. This evidence suggests that peer support may bolster confidence by counteracting the private interpretation of struggle as an individual failure. When NGNs recognized that uncertainty, stress, and gradual skill development were common features of the transition, they were better positioned to remain engaged in learning rather than to withdraw from practice situations that challenged their confidence.

Constructive feedback was another central support mechanism. Feedback from mentors, preceptors, peers, and team members helped NGNs calibrate their self‐assessment by identifying strengths, correcting errors, and clarifying expectations for safe practice [11, 24, 34, 37–41]. The synthesis suggests that feedback was most useful when it was specific, timely, behavior‐focused, and delivered within a trusting relationship. In contrast, vague criticism or dismissive responses could undermine confidence by increasing anxiety and discouraging NGNs from seeking help. Feedback, therefore, served both an educational and affective function: It helped NGNs evaluate their clinical performance while also reinforcing that learning was expected and supported during the transition period.

Overall, support contributed to confidence development through three interrelated pathways: guided participation with more experienced clinicians, belonging and normalization through peer connection, and feedback that helped NGNs calibrate their developing practice. These findings support the need to distinguish mentorship, preceptorship, peer support, and feedback as related but separate subcomponents of the support domain. The evidence also indicates that support is strongly shaped by organizational conditions. Protected preceptor time, stable mentor assignments, psychologically safe communication norms, and manageable workloads appeared to determine whether support was consistently available and whether NGNs could use it to build confidence. Thus, support should be understood not only as an interpersonal strategy but also as a nursing management responsibility requiring deliberate unit design and leadership infrastructure.

### 3.4. Skill Development

The domain of skill development includes structured learning experiences that help NGNs turn theoretical knowledge into competent practice. Throughout the literature, deliberate practice through simulation, hands‐on training, and continuing education has been identified as key strategies for building clinical skills and confidence. This domain emphasizes that confidence increases not only with support but also through repeated skill use and opportunities to improve judgment in realistic, practice‐based settings.

Skill development contributed to confidence primarily through mastery experiences provided in programs: repeated practice, simulation, and progressive exposure to complex patient situations [6–11, 20, 26, 34, 36, 42–44]. These programs often included hands‐on workshops and simulations that allowed nurses to practice critical procedures in a controlled environment. The repeated practice of technical skills significantly enhanced competence and confidence, particularly in high‐stakes situations [7, 11, 20].

Simulation‐based training and hands‐on workshops effectively bridge the gap between theoretical knowledge and practical application. Included studies discussed using simulation exercises that replicated real‐life scenarios, providing NGNs the opportunity to hone their decision‐making skills in a safe environment [6, 41, 42, 44–48]. Simulation was particularly beneficial in preparing nurses for unexpected or complex clinical situations, with participants reporting increased confidence in handling such challenges [42, 44, 45].

Continuing education and professional development were important strategies for building confidence. Numerous included studies highlighted the role of ongoing education in keeping nurses up to date with the latest practices and technologies [8, 11, 20, 34, 36, 40, 43, 49]. Continuing education programs are essential for sustaining confidence, particularly as nurses assume more complex roles and responsibilities [36, 40, 43].

### 3.5. Reflection

Reflection contributed to confidence by helping NGNs interpret emotional arousal, uncertainty, and mistakes as learning cues rather than evidence of incapability. Structured debriefing, guided self‐assessment, and reflective discussion converted clinical exposure into usable confidence‐building feedback. Several studies discussed the role of self‐reflection in helping NGNs evaluate their performance and identify areas for improvement [19, 30, 39, 43, 44, 50–50]. The included articles suggested that self‐reflection is most effective when structured and guided, allowing nurses to critically analyze their actions and decisions [30, 44, 50].

Debriefing sessions were frequently used to provide a structured environment for reflection and discussion. Multiple studies highlighted how debriefing after clinical experiences helped nurses process their experiences, learn from mistakes, and build confidence in their decision‐making abilities [19, 33, 37, 40, 43, 49, 50]. These sessions were often led by experienced nurses or educators who could provide additional insights and guidance [37, 40, 49].

Self‐assessment was another indispensable aspect of the reflection process, allowing nurses to take ownership of their learning and development. Several studies indicated that regular self‐assessment helps nurses to monitor their progress and set goals for ongoing improvement [19, 37, 39, 40, 43, 44, 49]. This process was particularly valuable in fostering a growth mindset, where challenges are viewed as opportunities for learning rather than threats to confidence [39, 40, 43].

### 3.6. Integration

Integration appears to unfold over time: NGNs first seek safe participation, then gradually master recurring tasks, and finally develop a more credible professional voice within the team. This temporal pattern suggests that confidence develops through progressive legitimate participation, not only through individual skill acquisition. This domain centers on how NGNs strengthen their professional roles through task mastery, teamwork, and structured role transition. Integration represents the process of becoming part of the clinical community, where competence, collaboration, and confidence grow together. Across studies, successful integration was associated with mastering daily responsibilities, feeling accepted within the team, and gradually taking on more independence, all of which boost professional confidence and a sense of belonging.

Several studies emphasized mastering critical clinical tasks to build confidence [11, 12, 21, 25, 29, 33, 35, 38, 50]. When new nurses feel confident performing their daily responsibilities, they are more likely to trust their decision‐making and take on more complex tasks [11, 25, 35].

Workgroup integration was also highlighted as a significant factor in building confidence. Multiple articles discussed the role of socialization and team dynamics in helping new nurses feel accepted and valued within their teams [12, 21, 25, 29–31, 35, 38, 50]. A supportive team environment, where nurses can rely on each other for assistance and advice, is essential for building confidence, especially in the early stages of their careers [21, 29, 38].

Role transition strategies, such as extended orientation programs and gradual increases in responsibility, were also noted as effective methods for building confidence. Several articles described how structured role transition processes helped new nurses to acclimate to their professional roles without feeling overwhelmed [11, 12, 22, 23, 25, 27, 34]. These strategies often included shadowing experienced nurses, participating in interdisciplinary teams, and gradually assuming more complex patient care responsibilities [22, 25, 34].

### 3.7. Influence of Organizational Culture

The domain of organizational culture captures how the broader work environment shapes NGNs’ confidence and professional growth. Across studies, cultures that emphasize psychological safety, inclusivity, and continuous learning are consistently linked to greater confidence and engagement. A supportive organizational climate, where teamwork, respect, and open communication are prioritized, enables new nurses to thrive, while adverse environments marked by incivility or bullying hinder confidence and professional development.

Organizational culture played a pivotal role in shaping the confidence of NGNs. Several articles discussed how a positive organizational culture, characterized by support, inclusivity, and a focus on professional development, contributed to the confidence of new nurses [5, 12, 19, 22–24, 29, 35, 39, 41]. A culture that promotes continuous learning, recognizes achievements, and addresses challenges constructively helps foster confidence [19, 24, 25].

Positive work environments were also identified as crucial for building confidence. Several articles highlighted the importance of creating a work environment where new nurses feel safe to ask questions, seek help, and make mistakes [12, 27, 29, 34, 35, 38, 39, 41, 50]. Environments that promote open communication and teamwork have been shown to significantly enhance NGNs’ confidence, especially in high‐pressure settings [34, 35, 39].

Finally, addressing bullying and negative behaviors in the workplace was recognized as essential in maintaining a supportive environment in which new nurses can thrive. Multiple articles examined the harmful effects of workplace bullying on the confidence and well‐being of NGNs [12, 19, 22, 24, 30, 32, 38, 50]. Interventions to reduce bullying and promote a positive culture were crucial in enabling new nurses to build confidence without fearing negative repercussions [22, 24, 32, 50].

## 4. Discussion

This integrative review advances the transition‐to‐practice literature by explaining professional confidence as a context‐sensitive systems outcome rather than an individual trait. The evidence suggests that confidence develops when NGNs have access to four mutually reinforcing mechanisms: supportive feedback and role modeling, mastery‐oriented skill development, structured reflection, and progressive integration into the clinical team. The key domains, support, skill development, reflection, integration, and culture, highlight both similarities and differences with previous research on the transition from student to professional nurse. For example, the literature supports the vital role of mentorship and preceptorship in boosting NGNs’ confidence, and this review confirms their significance. Similar to earlier studies, such as those by Chandler [29] and Banks et al. [41], the results demonstrate that these supportive relationships are crucial for offering guidance, emotional support, and practical advice. These findings align with those of Chandler [29], who emphasized the role of mentorship in facilitating the transition from student to professional nurse. However, this review also expands the understanding of these relationships by emphasizing the need for structured, ongoing mentorship programs, a detail that was less explicitly discussed in earlier research.

A consistent pattern across studies is that contextual contingency matters: the same strategy (e.g., simulation or mentorship) yields larger, more lasting confidence gains when embedded in supportive cultures. For example, when novices are intentionally integrated, psychological safety is fostered, and workload permits guided practice [5–13].

In contrast to previous research, which primarily focused on the benefits of formal mentorship programs, this review also supported the importance of peer engagement. While the importance of peer networks has been recognized in studies such as those by Willman et al. [31] and Waddell et al. [34], this review stresses the role of peer support in fostering a sense of community and reducing feelings of isolation among NGNs. This finding adds to the existing literature by suggesting that peer support is not merely supporting but integral to building confidence, especially when formal mentorship is not immediately available.

Skills training, particularly through simulation‐based methods, remains a recognized building block for building confidence. The findings of this review support the conclusions of earlier studies, such as those by Oblea et al. [6] and Della Ratta [7], which identified simulation as a critical tool in preparing nurses for high‐stakes situations. However, this review provides a more nuanced understanding by categorizing training into targeted skills training and continuing education. While previous research often discussed these aspects separately, this review integrates them, highlighting the ongoing need for education to sustain and enhance confidence as NGNs’ transition into more complex roles.

The findings of this review contrast with earlier literature that sometimes viewed simulation‐based training as sufficient for building confidence. For example, while Standing et al. [45] emphasized the immediate benefits of simulation, this review suggests that simulation should be complemented with continuous professional development to maintain confidence over time. This finding challenges the notion that confidence can be fully established in a controlled environment, advocating a more holistic approach to training that includes initial simulations and ongoing education.

The literature emphasizes the importance of reflection, primarily through self‐reflection and debriefing. Studies, such as those by Kooker and Kamikawa [50] and Mangone et al. [33], have long demonstrated that structured reflection fosters critical thinking and confidence in nursing. This review confirms these findings and introduces the concept of structured self‐assessment as an integral part of the reflection process. While earlier studies often focused on debriefing as the primary reflective tool, this review highlights self‐assessment as equally important. This differs from research, such as that of Manias et al. [19], which placed less emphasis on self‐assessment. Including self‐assessment in this review suggests that NGNs benefit from regularly evaluating their progress and setting personal goals, fostering a growth mindset. This addition broadens the scope of reflection, supporting a more active role for nurses in their development.

The themes of task mastery and workgroup integration identified in this review are consistent with earlier findings in the literature. Studies by Tomietto et al. [12] and Gorman and McDowell [35] have emphasized the importance of mastering daily responsibilities in building confidence. This review reinforces these findings, suggesting that task mastery enables NGNs to trust their nursing abilities. However, this review extends the discussion by integrating the role of socialization within workgroups into the narrative of confidence‐building. While previous literature, such as Kowalski and Cross [38], has acknowledged the importance of team dynamics, this review emphasizes the need for a supportive team environment to cultivate confidence. This perspective aligns with, but also expands upon, earlier research, suggesting that socialization within the workgroup is both beneficial and essential for successfully integrating NGNs into clinical practice.

The findings related to organizational culture align with previous literature emphasizing the importance of a supportive work environment. Research by Tomietto et al. [12] and Gorman and McDowell [35] has consistently demonstrated that a positive organizational culture is essential for fostering confidence among NGNs. This review supports these findings and further accentuates the importance of addressing negative behaviors, such as bullying, which previous studies, including those by McLaughlin et al. [32], have identified as detrimental to confidence.

One area where this review differs from earlier literature is its focus on organizations’ proactive role in fostering a culture of continuous learning and professional growth. While earlier studies often discussed organizational culture broadly, this review makes a more specific call to action. Institutions must build a positive culture and actively maintain it through effective policies, practices, and leadership support. The findings of this integrative review advance current knowledge by presenting an integrated, multifactorial model of confidence development in NGNs. While previous studies have often examined strategies in isolation, this review integrates them into a comprehensive framework. Specifically, it emphasizes five interconnected domains: support, skill development, reflection, integration, and culture, each of which uniquely contributes to confidence‐building during the transition to acute care practice.

A key theoretical advancement in this review is framing organizational culture as a broad moderator that influences the effectiveness of all other areas. This approach extends beyond earlier research that viewed interventions and workplace factors as separate. Instead, we argue that organizational culture fundamentally shapes how mentorship, skills training, reflection, and integration contribute to building confidence. This system‐oriented perspective suggests that interventions may not be fully effective in unsupportive or unsafe work environments. Additionally, the review highlights dynamic, cyclical relationships among various areas, particularly between reflection and integration. Nurses who engage in structured reflection are more likely to integrate into workgroups and master tasks, thereby boosting their confidence and deepening their reflective insights. This demonstrates that confidence‐building is not a straightforward process but an ongoing cycle.

The findings should be interpreted with caution because not all included studies measured confidence directly. Studies using competence, readiness, job satisfaction, or transition shock were retained because they illuminated conditions related to confidence development, but they were treated as proxy evidence. Therefore, the model should be read as a theoretically informed synthesis of confidence‐building mechanisms rather than evidence that each strategy produces direct measurable increases in confidence across all contexts.

### 4.1. The Multifactorial Model of Confidence Development

**FIGURE 2:**
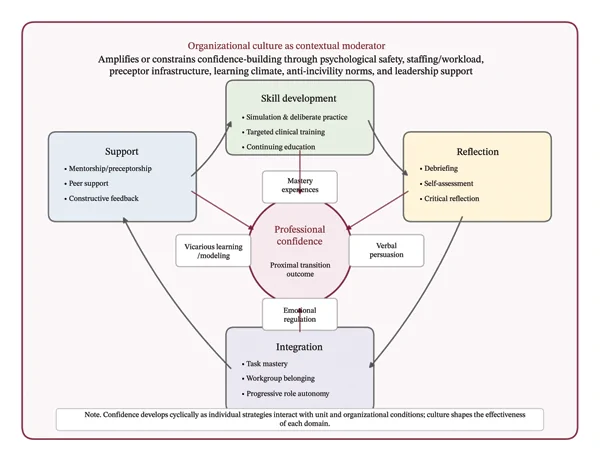
Multifactorial model of professional confidence development in newly graduated nurses.

Figure 2 presents the multifactorial model of professional confidence development. The model distinguishes the major domains and subcomponents identified in the synthesis while showing organizational culture as the contextual condition that shapes whether these strategies translate into durable confidence gains. The model illustrates a cyclical, dynamic process in which mentorship and structured support foster skill development [6, 25], which, in turn, enables meaningful reflection through self‐assessment and debriefing [43, 45]. Reflective insights drive integration into workgroups and mastery of tasks [19, 33], with successful integration facilitating reciprocal support and peer mentoring [12, 31]. Workplace culture serves as a central moderator, amplifying or constraining each process by influencing psychological safety, professional development, and team cohesion [24, 35].

### 4.2. Implications

The findings of this review hold implications for nursing practice, policy, and future research. For practice, the findings emphasize the importance of implementing structured mentorship and preceptorship programs, simulation‐based training, and fostering a supportive organizational culture to effectively build NGNs’ confidence. From a policy perspective, the variability in the quality of confidence‐building interventions across healthcare settings underscores the need for standardized guidelines to ensure consistent support for NGNs. Future research should focus on conducting HQ, quantitative studies to evaluate the long‐term effectiveness of these strategies and explore their applicability in diverse healthcare contexts, including low‐ and middle‐income countries. This review suggests that a comprehensive, multifaceted approach is essential to optimizing the transition of NGNs into confident, competent practitioners. The conceptual framework developed in this review also provides a foundation for the development of middle‐range theories in nursing education and workforce research.

Health systems should treat confidence as a systems outcome and implement structural supports: (1) protected preceptor time and continuity; (2) standardized, staged socialization with clear role expectations in Months 0–6 and 6–12; (3) workload and staffing that permit deliberate practice; and (4) routines that build psychological safety (e.g., debriefing and just culture). Under these conditions, individual‐level strategies (simulation and targeted skills training) translate into durable confidence gains [5–8], aligning confidence with retention‐relevant pathways [11–13]. Specifically for nurse managers, the practical implication is that confidence‐building requires unit design. Protected preceptor time, preceptor continuity, gradual autonomy, workload that permits questions and supervised practice, routine debriefing, and visible anti‐incivility norms are not optional supports; they are mechanisms through which confidence‐building interventions become effective.

### 4.3. Strengths and Limitations

This review has several limitations. First, the variability in the methodological quality of the included studies, particularly those relying on self‐reported measures of confidence, may affect the reliability of the findings. The predominance of qualitative studies limits the ability to draw definitive conclusions about the efficacy of specific interventions. Additionally, the review’s focus on studies conducted in high‐income countries restricts the generalizability of the findings to other healthcare contexts. The exclusion of non‐English‐language studies and potential studies missed outside the searched databases may have introduced bias and limited the scope of the review. Finally, while necessary for focus, the inclusion criteria may have excluded relevant perspectives from more experienced nurses or nonacute care settings, potentially overlooking alternative strategies for building confidence. The strengths of this review include a thorough literature search employing a comprehensive methodology focused on reducing search bias, resulting in a scholarly piece that can inform and enhance the transition stage for NGNs. Additional limitations include the absence of a registered protocol, the English‐language and full‐text limits, exclusion of gray literature, and heterogeneity in how confidence was operationalized. Because several studies used proxy outcomes, causal claims about confidence development should be avoided.

Although this review focuses on confidence as a proximal outcome, it is recognized that emphasizing confidence alone may be misconstrued as attributing systemic issues to individual shortcomings. Several studies have focused on individual strategies; future research should prioritize meso‐ and macrolevel interventions (e.g., staffing models, preceptor workload protection, and unit culture) to examine confidence as a context‐dependent outcome.

## 5. Conclusions

Professional confidence among NGNs develops through a dynamic interaction between individual learning experiences and organizational conditions. Mentorship, simulation, reflection, and team integration are most likely to build confidence when embedded in psychologically safe and structurally supportive units. Future research should directly measure confidence/self‐efficacy, test the proposed mechanisms, and examine how organizational culture conditions transition outcomes.

## Author Contributions

Justin Fontenot: conceptualization, writing, editing, data collection, data analysis, search strategy, and search. Fuqin Liu: conceptualization, search strategy, search selection, data analysis, editing, and manuscript writing. Jincy Immanuel: conceptualization, search strategy, editing, and manuscript writing.

## Funding

No funding (either private or public) was used for this literature review. This research received no specific grant from any funding agency in the public, commercial, or not‐for‐profit sectors.

## Disclosure

All regenerated text was checked for accuracy, and the authors are accountable for all content in this manuscript.

## Ethics Statement

No patients or any other human subjects were included in this literature review.

## Consent

The authors have nothing to report.

## Conflicts of Interest

The authors declare no conflicts of interest.

## Supporting Information

Additional supporting information can be found online in the Supporting Information section.

## Supporting information


**Supporting Information** Table S1 is a supporting file that includes the nature of the evidence from each study and how the evidence was used in the synthesis for this integrative review. Table S1: Study Characteristics, evidence tier, domain contribution, and quality appraisal for included studies.

## Data Availability

This paper reports the findings of a literature review; therefore, no dataset is associated with this work. Those who wish to review the articles can repeat the search using the search details provided in Table 1.

## References

[1] Baker O. G. , New Graduate Nurses’ Transition: Role of Lack of Experience and Knowledge as Challenging Factors, Saudi Journal for Health Sciences. (2020) 9, no. 3, 214–220, 10.4103/sjhs.sjhs_88_20.

[2] Xu G. , Zeng X. , and Wu X. , Global Prevalence of Turnover Intention Among Intensive Care Nurses: A Meta‐Analysis, Nursing in Critical Care. (2023) 28, no. 2, 159–166, 10.1111/nicc.12679. 34261191

[3] United States Bureau of Labor Statistics , Registered Nurses: Occupational Outlook Handbook, 2025, https://www.bls.gov/ooh/healthcare/registered-nurses.htm#tab-6.

[4] Nsi Nursing Solutions , 2025 NSI National Health Care Retention & RN Staffing Report, 2025, https://www.nsinursingsolutions.com/documents/library/nsi_national_health_care_retention_report.pdf.

[5] Charette M. , McKenna L. , McGillion A. , and Burke S. , Effectiveness of Transition Programs on New Graduate Nurses’ Clinical Competence, Job Satisfaction and Perceptions of Support: a Mixed‐Methods Study, Journal of Clinical Nursing. (2023) 32, no. 7/8, 1354–1369, 10.1111/jocn.16317. 35451137

[6] Oblea P. N. , Berry-Caban C. S. , Dumayas J. Y. , Adams A. R. , and Beltran T. A. , Evaluation of Clinical Nurse Transition Program at US Army Hospitals, Military Medicine. (2019) 184, no. 11–12, 914–921, 10.1093/milmed/usz108. 31067330

[7] Della Ratta C. , Challenging Graduate Nurses’ Transition: Care of the Deteriorating Patient, Journal of Clinical Nursing. (2016) 25, no. 19–20, 3036–3048, 10.1111/jocn.13358. 27524113

[8] Zamanzadeh V. , Roshangar F. , Fathi-Azar E. , Valizadeh L. , and Kirkwood J. , Experiences of Newly Graduated Nurses on Strategies of Gaining Self-Confidence During Their Initial Work: A Qualitative Study, Journal of Nursing Research. (2014) 22, no. 4, 283–291, 10.1097/jnr.0000000000000050. 25265368

[9] Grubaugh M. L. , Africa L. , and Loresto F. L. , Exploring the Relationship between Psychological Capital and Turnover Among New Nurses, Nurse Leader. (2023) 21, no. 3, 409–414, 10.1016/j.mnl.2023.02.002. 37274757 PMC9970916

[10] Wang X. , Liu M. , Xu T. , Wang K. , Huang L. , and Zhang X. , New Nurses’ Practice Environment, Job Stress, and Patient Safety Attitudes: A Cross-Sectional Study Based on the Job Demands-Resources Model, BMC Nursing. (2024) 23, no. 1, 10.1186/s12912-024-02135-0. PMC11241995 38997677

[11] Tong Y. , Wang T. , Tong S. et al., Relationship Among Core Competency, Self-Efficacy and Transition Shock in Chinese Newly Graduated Nurses: A Cross-Sectional Study, BMJ Open. (2024) 14, no. 4, 10.1136/bmjopen-2023-082865. PMC11146377 38569675

[12] Tomietto M. , Rappagliosi C. M. , Sartori R. , and Battistelli A. , Newcomer Nurses’ Organisational Socialisation and Turnover Intention During the First 2 Years of Employment, Journal of Nursing Management. (2015) 23, no. 7, 851–858, 10.1111/jonm.12224. 24635003

[13] Labrague L. J. and De los Santos J. A. A. , Transition Shock and Newly Graduated Nurses’ Job Outcomes and Select Patient Outcomes: A Cross‐Sectional Study, Journal of Nursing Management. (2020) 28, no. 5, 1070–1079, 10.1111/jonm.13033. 32315478

[14] Shorey S. and Lopez V. , Haugan G. and Eriksson M. , Self-Efficacy in a Nursing Context, Health Promotion in Health Care–Vital Theories and Research, 2021, Springer International Publishing, 145–158, 10.1007/978-3-030-63135-2_12.

[15] Tomita R. , The Relationship Between General Self‐Efficacy and Nursing Practice Competence for Second‐Year Nurses: Empirical Quantitative Research, Nursing Open. (2024) 11, no. 7, 10.1002/nop2.2233. PMC11222662 38961662

[16] Bandura A. , Self-Efficacy: Toward a Unifying Theory of Behavioral Change, Psychological Review. (1977) 84, no. 2, 191–215, 10.1037/0033-295X.84.2.191. 847061

[17] Whittemore R. and Knafl K. , The Integrative Review: Updated Methodology, Journal of Advanced Nursing. (2005) 52, no. 5, 546–553, 10.1111/j.1365-2648.2005.03621.x. 16268861

[18] JBI , Quality Appraisal Tools, 2025, https://jbi.global/critical-appraisal-tools.

[19] Manias E. , Aitken R. , and Dunning T. , Decision-Making Models Used by “Graduate Nurses” Managing Patients’ Medications, Journal of Advanced Nursing. (2004) 47, no. 3, 270–278, 10.1111/j.1365-2648.2004.03091.x. 15238121

[20] Olson M. E. , The “Millennials”: First Year in Practice, Nursing Outlook. (2009) 57, no. 1, 10–17, 10.1016/j.outlook.2008.06.001. 19150262

[21] Ke Y. and Stocker J. F. , On the Difficulty of Finding One’s Place: A Qualitative Study of New Nurses’ Processes of Growth in the Workplace, Journal of Clinical Nursing. (2019) 28, no. 23/24, 4321–4331, 10.1111/jocn.14996. 31294495

[22] Lusk Monagle J. , Lasater K. , Stoyles S. , and Dieckmann N. , New Graduate Nurse Experiences in Clinical Judgment: What Academic and Practice Educators Need to Know, Nursing Education Perspectives. (2018) 39, no. 4, 201–207, 10.1097/01.NEP.0000000000000336. 29746356

[23] Doughty L. , Sinnema C. , McKillop A. , and Dixon R. , The Impact of Postgraduate Education in Transition to Practice Programmes on New Graduate Nurses’ Knowledge and Skills: A Pre-Post Survey Design, Nurse Education Today. (2021) 102, 10.1016/j.nedt.2021.104888. 33894592

[24] Gazaway S. , Gibson R. W. , Schumacher A. , and Anderson L. , Impact of Mentoring Relationships on Nursing Professional Socialization, Journal of Nursing Management. (2019) 27, no. 6, 1182–1189, 10.1111/jonm.12790. 31099912

[25] Charette M. , McKenna L. , McGillion A. , and Burke S. , Effectiveness of Transition Programs on New Graduate Nurses’ Clinical Competence, Job Satisfaction and Perceptions of Support: A Mixed‐Methods Study, Journal of Clinical Nursing. (2022) 32, no. 7-8, 1354–1369, 10.1111/jocn.16317. 35451137

[26] Byermoen K. R. , Brembo E. A. , Egilsdottir H. Ö. et al., Newly Graduated Nurses Use and Further Development of Assessment skills—An In‐Depth Qualitative Study, Journal of Advanced Nursing. (2023) 79, no. 9, 3286–3298, 10.1111/jan.15631. 36876732

[27] Cubit K. and Lopez V. , Qualitative Study of Enrolled Nurses Transition to Registered Nurses, Journal of Advanced Nursing. (2012) 68, no. 1, 206–211, 10.1111/j.1365-2648.2011.05729.x. 21668481

[28] Allan H. T. , Magnusson C. , Evans K. et al., Putting Knowledge to Work in Clinical Practice: Understanding Experiences of Preceptorship as Outcomes of Interconnected Domains of Learning, Journal of Clinical Nursing. (2018) 27, no. 1–2, 123–131, 10.1111/jocn.13855. 28401608

[29] Chandler G. E. , Succeeding in the First Year of Practice: Heed the Wisdom of Novice Nurses, Journal for Nurses in Staff Development: JNSD: Official Journal of the National Nursing Staff Development Organization. (2012) 28, no. 3, 103–107, 10.1097/NND.0b013e31825514ee. 22617780

[30] Devey Burry R. , Stacey D. , Backman C. , Donia M. B. L. , and Lalonde M. , Exploring Pairing of New Graduate Nurses With Mentors: An Interpretive Descriptive Study, Journal of Clinical Nursing. (2020) 29, no. 15/16, 2897–2906, 10.1111/jocn.15360. 32497315

[31] Willman A. , Bjuresäter K. , and Nilsson J. , Insufficiently Supported in Handling Responsibility and Demands: Findings From a Qualitative Study of Newly Graduated Nurses, Journal of Clinical Nursing. (2021) 30, no. 1–2, 83–92, 10.1111/jocn.15483. 32889729 PMC7891354

[32] McLaughlin J. L. , Reed J. A. , Shiveley J. , and Lee S. , Escape Room Blueprint: Central Orientation Contagion Crisis, Simulation & Gaming. (2021) 52, no. 1, 24–30, 10.1177/1046878120954493.

[33] Mangone N. , King J. , Croft T. , and Church J. , Group Debriefing: An Approach to Psychosocial Support for New Graduate Registered Nurses and Trainee Enrolled Nurses, Contemporary Nurse. (2005) 20, no. 2, 248–257, 10.5172/conu.20.2.248. 16393106

[34] Waddell J. , Spalding K. , Navarro J. , Jancar S. , and Canizares G. , Integrating a Career Planning and Development Program Into the Baccalaureate Nursing Curriculum. Part II. Outcomes for New Graduate Nurses 12 Months Post-Graduation, International Journal of Nursing Education Scholarship. (2015) 12, no. 1, 175–182, 10.1515/ijnes-2015-0028. 26618574

[35] Gorman L. L. and McDowell J. R. S. , Identifying the Needs of Critical and Acute Cardiac Care Nurses Within the First Two Years of Practice in Egypt Using a Nominal Group Technique, Nurse Education in Practice. (2018) 28, no. 101090848, 127–134, 10.1016/j.nepr.2017.10.005. 29078108

[36] Feltrin C. , Newton J. M. , and Willetts G. , How Graduate Nurses Adapt to Individual Ward Culture: A Grounded Theory Study, Journal of Advanced Nursing. (2019) 75, no. 3, 616–627, 10.1111/jan.13884. 30375010

[37] Ten Hoeve Y. , Kunnen S. , Brouwer J. , and Roodbol P. F. , The Voice of Nurses: Novice Nurses’ First Experiences in a Clinical Setting A Longitudinal Diary Study, Journal of Clinical Nursing. (2018) 27, no. 7–8, e1612–e1626, 10.1111/jocn.14307. 29446496

[38] Kowalski S. and Cross C. L. , Preliminary Outcomes of a Local Residency Programme for New Graduate Registered Nurses, Journal of Nursing Management. (2010) 18, no. 1, 96–104, 10.1111/j.1365-2834.2009.01056.x. 20465735

[39] Aggar C. , Bloomfield J. , Thomas T. H. , and Gordon C. J. , Australia’s First Transition to Professional Practice in Primary Care Program for Graduate Registered Nurses: A Pilot Study, BMC Nursing. (2017) 16, 1–11, 10.1186/s12912-017-0207-5. 28344514 PMC5363053

[40] Lindfors K. , Flinkman M. , Kaunonen M. , Huhtala H. , and Paavilainen E. , New Graduate Registered Nurses’ Professional Competence and the Impact of Preceptors’ Education Intervention: A Quasi-Experimental Longitudinal Intervention Study, BMC Nursing. (2022) 21, no. 1, 1–12, 10.1186/s12912-022-01133-4. 36526985 PMC9757917

[41] Banks P. , Roxburgh M. , Kane H. et al., Flying Start NHS™: Easing the Transition From Student to Registered Health Professional, Journal of Clinical Nursing. (2011) 20, no. 23–24, 3567–3576, 10.1111/j.1365-2702.2011.03796.x. 21831108

[42] Lim S. H. , Ang S. Y. , Aloweni F. , Siow K. C. E. , Koh S. B. L. , and Ayre T. C. , Factors Associated With Practice Readiness Among Newly Qualified Nurses in Their First Two Years of Practice, Nurse Education Today. (2024) 136, 10.1016/j.nedt.2024.106143. 38422796

[43] Hussein R. , Everett B. , Ramjan L. M. , Hu W. , and Salamonson Y. , New Graduate Nurses’ Experiences in a Clinical Specialty: A Follow up Study of Newcomer Perceptions of Transitional Support, BMC Nursing. (2017) 16, 1–9, 10.1186/s12912-017-0236-0. 28775671 PMC5534089

[44] Cleary M. , Horsfall J. , Mannix J. , O’Hara‐Aarons M. , and Jackson D. , Valuing Teamwork: Insights From Newly‐Registered Nurses Working in Specialist Mental Health Services, International Journal of Mental Health Nursing. (2011) 20, no. 6, 454–459, 10.1111/j.1447-0349.2011.00752.x. 21676136

[45] Standing M. , Clinical Decision-Making Skills on the Developmental Journey From Student to Registered Nurse: A Longitudinal Inquiry, Journal of Advanced Nursing. (2007) 60, no. 3, 257–269, 10.1111/j.1365-2648.2007.04407.x. 17850295

[46] Kaddoura M. A. , New Graduate Nurses’ Perceptions of the Effects of Clinical Simulation on Their Critical Thinking, Learning, and Confidence, The Journal of Continuing Education in Nursing. (2010) 41, no. 11, 506–516, 10.3928/00220124-20100701-02. 20672760

[47] Murray M. , Sundin D. , and Cope V. , A Mixed-Methods Study on Patient Safety Insights of New Graduate Registered Nurses, Journal of Nursing Care Quality. (2020) 35, no. 3, 258–264, 10.1097/NCQ.0000000000000443. 32433150

[48] AbuAlRub R. F. and Abu Alhaija’a M. G. , Perceived Benefits and Barriers of Implementing Nursing Residency Programs in Jordan, International Nursing Review. (2019) 66, no. 1, 43–51, 10.1111/inr.12452. 29498034

[49] Patterson E. E. B. , Boyd L. , and Mnatzaganian G. , The Impact of Undergraduate Clinical Teaching Models on the Perceptions of Work-Readiness Among New Graduate Nurses: A Cross Sectional Study, Nurse Education Today. (2017) 55, 101–106, 10.1016/j.nedt.2017.05.010. 28575706

[50] Kooker B. M. and Kamikawa C. , Successful Strategies to Improve RN Retention and Patient Outcomes in a Large Medical Centre in Hawaii, Journal of Clinical Nursing. (2011) 20, no. 1–2, 34–39, 10.1111/j.1365-2702.2010.03476.x. 21054602

[51] Charette M. , Goudreau J. , and Bourbonnais A. , Factors Influencing the Practice of New Graduate Nurses: A Focused Ethnography of Acute Care Settings, Journal of Clinical Nursing. (2019) 28, no. 19–20, 3618–3631, 10.1111/jocn.14959. 31190368

