# Supplementary material for: A Multifactorial Model for Building Professional Confidence in New Graduate Nurses: Integrative Review of Influential Strategies and Contexts
Source: J Nurs Manag. 2026 Aug 23;2026:6942823. doi: 10.1155/jonm/6942823 (PMC13501013; doi:10.1155/jonm/6942823)
Supplement: Supplementary file 1 [file JONM-2026-6942823-s001.docx]

**Supplementary Table S1**

**Table S1**

*Study Characteristics, Evidence Tier, Domain Contribution, and Quality Appraisal for Included Studies*

| **Study** | **Country/region** | **Design and sample** | **Evidence tier** | **Domain contribution** | **Quality** | **Key finding** | **Use in synthesis** |
| --- | --- | --- | --- | --- | --- | --- | --- |
| Tomietto et al. (2015) | Italy | Cross-sectional; N = 156 | Related/proxy | Integration; organizational culture | High | Organizational socialization and newcomer stage influenced turnover intention across the first 2 years. | Supports culture/socialization as a contextual condition rather than a stand-alone intervention. |
| Willman et al. (2020) | Sweden | Qualitative; N = 16 | Related/proxy | Support; integration | High | NGRNs described loneliness, high demands, and insufficient support in complex patient situations. | Supports peer/team support and belonging as conditions for confidence development. |
| Oblea et al. (2019) | Multinational | Prospective pre-/post-test evaluation; N = 86 | Related/proxy | Support; skill development | Moderate | Transition program improved readiness, competency, clinical skills, teamwork, and communication. | Use as supporting evidence for structured transition programming; avoid treating as direct confidence evidence unless measured directly in the study. |
| McLaughlin et al. (2020) | United States | Observational; N = 46 | Direct confidence | Support; skill development | High | Escape-room activity was associated with satisfaction and confidence applying skills to the clinical role. | Supports simulation/game-based learning as mastery experience; interpret cautiously due to design. |
| Mangone et al. (2005) | Australia | Qualitative focus groups; N = 15 | Related/proxy | Support; reflection | High | Group debriefing provided psychosocial support and reduced reality shock. | Supports debriefing as reflection and peer normalization mechanism. |
| ten Hoeve et al. (2024) | Netherlands | Longitudinal qualitative IPA; N = 18 | Related/proxy | Support; reflection; integration | High | Relatedness, competence, and shared experience with colleagues shaped transition experiences. | Supports belonging and guided meaning-making as mechanisms in the model. |
| Tong et al. (2024) | China | Cross-sectional observational; N = 262 | Direct self-efficacy | Skill development; organizational culture | High | Self-efficacy mediated the relationship between core competency and transition shock. | Key evidence for theoretical grounding in self-efficacy and the link between confidence-related constructs and transition shock. |
| Standing (2007) | United Kingdom | Observational/retrospective interviews; N = 20 | Direct confidence | Support; reflection; skill development | High | Supportive mentors, reflective models, clinical science, and prior experiences improved confidence; stress and lack of resources decreased confidence. | Strong support for multi-source confidence development and context sensitivity. |
| Murray et al. (2020) | Australia | Longitudinal mixed methods; N = 210 | Related/proxy | Skill development; reflection | High | New graduates reported changing knowledge of medical errors and challenges with safety, time management, mistakes, experiential learning, and transition. | Use mainly for patient-safety learning and reflective experience; not direct confidence evidence. |
| Manias et al. (2004) | Australia | Qualitative observational; N = 12 | Related/proxy | Reflection; integration | High | Educational strategies supported cue use and medication-management recommendations in contextual practice. | Supports clinical reasoning, cue recognition, and practice-context learning. |
| Lim et al. (2024) | Singapore | Cross-sectional; N = 445 | Related/proxy | Skill development | High | Newly qualified nurses identified skills they were uncomfortable performing independently and concerns about competence/readiness. | Supports skill-specific discomfort as a confidence-adjacent management target. |
| Kowalski & Cross (2010) | United States | Observational residency evaluation; N = 55 | Related/proxy | Integration; organizational culture | Unclear | Residency program improved competency, sense of threat, communication, and leadership skills. | Use cautiously due to appraisal uncertainty; supports residency as a contextual intervention. |
| Waddell et al. (2015) | United States | Randomized controlled trial; N = 9 | Related/proxy | Support; reflection | High | Structured CPD supported career resilience and job satisfaction. | Use as limited-sample evidence for reflective/professional development processes. |
| Kooker & Kamikawa (2011) | United States | Longitudinal observational; participant count not reported | Related/proxy | Organizational culture | Unclear | Magnet pilot units were associated with improved retention over time. | Use as contextual management evidence, not direct confidence evidence. |
| Patterson et al. (2019) | Australia | Cross-sectional; N = 28 | Related/proxy | Skill development; reflection | High | University Fellowship Program was associated with increased work-readiness perceptions. | Supports structured academic-practice transition models; proxy for confidence. |
| Hussein et al. (2017) | Australia | Longitudinal convergent mixed methods; N = 87 | Related/proxy | Reflection; skill development | High | No significant changes in satisfaction with transitional support over 12 months. | Important contradictory/neutral finding; include to avoid overclaiming effectiveness. |
| Gorman & McDowell (2018) | Egypt | Qualitative descriptive/nominal group; N = 11 | Related/proxy | Integration; organizational culture | High | Identified professional needs of new acute cardiac nurses; organizational structure emerged as a distinctive theme. | Supports international/contextual variation in transition needs. |
| Gazaway et al. (2019) | United States | Grounded theory; N = 7 | Direct confidence | Support; integration; organizational culture | High | Mentoring provided emotional support, acceptance, guidance, and encouragement; mentors helped NGNs assimilate knowledge and gain confidence. | Strong qualitative evidence for mentorship mechanism and professional socialization. |
| Olson (2009) | United States | Longitudinal qualitative phenomenology; N = 12 | Related/proxy | Support; integration | High | Orientation and preceptorship were important in understanding millennial novice nurses’ first-year perceptions. | Supports structured orientation/preceptorship and need for evaluation. |
| Ke & Stocker (2019) | Taiwan | Exploratory qualitative content analysis; N = 20 | Related/proxy | Integration | Unclear | New nurses moved through disillusionment/shock, experiential knowledge, and finding a place for oneself. | Supports temporal staging and integration process; use cautiously due to appraisal uncertainty. |
| Lusk Monagle et al. (2018) | United States | Mixed-methods randomized pre-/post-test; N = 74 | Related/proxy | Reflection | High | Structured reflection had positive impact despite no significant quantitative group differences. | Important mixed finding; supports reflection while tempering causal claims. |
| Zamanzadeh et al. (2014) | Iran | Qualitative content analysis; N = 30 | Direct confidence | Support; skill development; reflection | High | NGNs used familiarity, knowledge enhancement, skills learning, self-exploration, validation, responsibility, interaction, and creativity to gain self-confidence. | Key direct evidence for confidence-building strategies and multiple self-efficacy sources. |
| Doughty et al. (2021) | New Zealand | Two-group pre-/post-test observational intervention; N = 85 | Related/proxy | Skill development; support | Moderate | Postgraduate course in a new graduate entry-to-practice program improved knowledge and skills. | Supports structured education as proxy evidence; not direct confidence unless separately measured. |
| Aggar et al. (2017) | Australia | Longitudinal exploratory mixed methods; N = 6 | Related/proxy | Support; integration | High | Graduate nurses were found competent in first-year general practice transition. | Use cautiously for acute-care relevance if retained; primarily competence evidence. |
| Charette et al. (2023) | Australia | Longitudinal mixed methods; N = 278 | Related/proxy | Skill development; support | High | Competence increased over 3 months and participants transitioned out of student mode. | Strong transition-program evidence, but mainly competence rather than direct confidence. |
| Della Ratta (2016) | United States | Qualitative IPA; N = 8 | Direct/related | Skill development; support; integration | High | Trusted relationships and caring for deteriorating patients shaped uncertainty, identity transformation, and self-understanding. | Supports high-stakes mastery, trusted backup, and identity transition. |
| Cleary et al. (2011) | Australia | Qualitative interviews; N = 13 | Related/proxy | Skill development; reflection; integration | High | Newly registered nurses valued teamwork, self-development, experiential learning, and positive patient engagement. | Supports teamwork and experiential learning as confidence-adjacent processes. |
| Byermoen et al. (2023) | Norway | Phenomenological-hermeneutical qualitative; N = 8 | Related/proxy | Support; integration; skill development | High | Relationship building, communication, context, recognition, and support influenced assessment-skill development. | Supports recognition and contextual learning in assessment confidence. |
| Cubit & Lopez (2012) | Australia | Qualitative focus groups; N = 10 | Related/proxy | Support; integration | High | Transition from enrolled nurse to registered nurse required specific support and recognition of prior experience. | Use as role-transition support evidence if population fit is justified. |
| Devey Burry et al. (2020) | Canada | Interpretive descriptive qualitative; N = 31 | Direct/related | Support; organizational culture | High | Mentor-mentee pairing was shaped by resources, scheduling, workload, unit dynamics, and registration-exam issues affecting confidence. | Strong support for organizational conditions shaping mentorship effectiveness. |
| Lindfors et al. (2022) | Finland | Quasi-experimental longitudinal intervention; N = 114 | Related/proxy | Skill development; support | Moderate | Competence improved in both groups over 9 months after a preceptor education intervention. | Supports preceptor development and competence gains; interpret as proxy confidence evidence. |
| Charette et al. (2022) | Australia | Longitudinal mixed methods; N = 150 | Direct/related | Skill development; support; integration | High | Competence increased over 3, 6, and 12 months; participants felt more confident and in control at 6 months. | Key evidence bridging competence and confidence over time. |
| Chandler (2012) | United States | Qualitative descriptive; N = 36 | Related/proxy | Support; organizational culture | High | Supportive, inclusive environments, positive relationships, and constructive feedback supported first-year success. | Supports feedback, inclusion, and culture as management levers. |
| Feltrin et al. (2019) | Australia | Constructivist grounded theory; N = 7 | Related/proxy | Integration; organizational culture | High | Graduate nurses adapted to individual ward cultures through self-embodiment, self-consciousness, navigating social constructs, and raising consciousness. | Strong evidence for culture as local ward-level conditioning context. |
| Charette et al. (2019) | Canada | Focused ethnography; N = 19 | Related/proxy | Skill development; integration; organizational culture | High | Competency deployment was influenced by orientation, workload, personality, and previous placements. | Strong evidence for conditions that enable or constrain competence enactment. |
| Banks et al. (2011) | Scotland | Descriptive mixed methods; N = 547 | Direct/related | Support; skill development | Unclear | Flying Start NHS positively affected clinical skills development and confidence, with greater satisfaction in community settings. | Large sample and direct confidence mention, but use cautiously due to unclear appraisal and setting variation. |
| Baumann et al. (2019) | Canada | Qualitative exploratory; N = 41 | Related/proxy | Integration; organizational culture | Moderate | Extended orientation supported work readiness and specialty-practice integration, with consistency challenges. | Supports orientation infrastructure and specialty context. |
| AbuAlRub & Abu Alhaija'a (2019) | Jordan | Descriptive qualitative; N = 36 | Related/proxy | Support; skill development; organizational culture | High | Identified barriers and perceived benefits of nursing residency programs for novice nurses. | Supports international relevance and implementation barriers. |
| Allan et al. (2018) | England | Ethnographic case study; N = 101 | Related/proxy | Support; integration | High | Preceptorship supported transition, adjustment, and competence development through interconnected learning domains. | Strong evidence for preceptorship and sociocultural integration. |
| Kaddoura (2010) | United States | Qualitative simulation study; N = 10 | Direct confidence | Skill development; reflection | High | Clinical simulation promoted critical thinking, learning, leadership, communication, reflection, and confidence in critical care training. | Key direct evidence for simulation as mastery, reflection, and self-efficacy support. |

*Note. Direct confidence = confidence/self-confidence/professional confidence/self-efficacy was explicitly measured or reported. Direct/related = confidence was reported alongside competence, readiness, or other transition outcomes. Related/proxy = confidence was inferred from adjacent outcomes such as competence, work readiness, satisfaction, socialization, retention, transition shock, skill performance, or perceived support. Quality ratings should be aligned with the specific JBI critical appraisal checklist used for each design. If retained in the manuscript, the methods should specify which JBI checklist was used for qualitative, cross-sectional, quasi-experimental, randomized, cohort/longitudinal, and mixed-methods studies, and how ratings were converted into High, Moderate, Low, or Unclear categories.*
